# Electrolyte contact changes nano-Li_4_Ti_5_O_12_ bulk properties via surface polarons

**DOI:** 10.1038/s42004-023-00913-6

**Published:** 2023-06-07

**Authors:** P. Philipp M. Schleker, Cristina Grosu, Marc Paulus, Peter Jakes, Robert Schlögl, Rüdiger-A. Eichel, Christoph Scheurer, Josef Granwehr

**Affiliations:** 1 Institut für Grundlagen der Elektrochemie IEK-9, Forschungszentrum Jülich, Wilhelm-Johnen Straße, 52425 Jülich, Germany; 2 Institut für Chemie, Technische Universität München, 85748 Garching b, München, Germany; 3 Institut für Physikalische Chemie (IPC), RWTH Aachen University, D-52074 Aachen, Germany; 4 Fritz-Haber-Institut der Max-Planck-Gesellschaft Faradayweg 4–6, 14195 Berlin, Germany; 5 Institut für Technische und Makromolekulare Chemie (ITMC), RWTH Aachen University, D-52074 Aachen, Germany

**Keywords:** Solid-state NMR, Batteries, Surfaces, interfaces and thin films

## Abstract

It is of general interest to combine the faradaic processes based high energy density of a battery with the non-faradaic processes based high power density of a capacitor in one cell. Surface area and functional groups of electrode materials strongly affect these properties. For the anode material Li_4_Ti_5_O_12_ (LTO), we suggest a polaron based mechanism that influences Li ion uptake and mobility. Here we show electrolytes containing a lithium salt induce an observable change in the bulk NMR relaxation properties of LTO nano particles. The longitudinal ^7^Li NMR relaxation time of bulk LTO can change by almost an order of magnitude and, therefore, reacts very sensitively to the cation and its concentration in the surrounding electrolyte. The reversible effect is largely independent of the used anions and of potential anion decomposition products. It is concluded that lithium salt containing electrolytes increase the mobility of surface polarons. These polarons and additional lithium cations from the electrolyte can now diffuse through the bulk, induce the observed enhanced relaxation rate and enable the non-faradaic process. This picture of a Li^+^ ion equilibrium between electrolyte and solid may help with improving the charging properties of electrode materials.

## Abstract (web summary)

The surface chemistries of electrode materials strongly influence their faradaic and non-faradaic properties. Here, the anode material Li_4_Ti_5_O_12_ is probed in the presence of different electrolytes, and surface polarons are suggested to enable a Li ion equilibrium between the bulk and the electrolyte, explaining the role of defects for intercalative pseudocapacity and fast charging properties in this material.

## Introduction

Energy storage systems are essential for a broad range of applications with requirements as diverse as powering a continuously increasing number of portable devices, providing traction power in electrically powered vehicles, or stabilizing the power level in electricity networks. Therefore, industrially viable materials for energy storage systems need to be adapted to various use cases^1^, which requires detailed mechanistic understanding of underlying processes in batteries and capacitors^2,3^. The energy density and the power density are two essential parameters, the former describing the amount of energy that can be stored and the latter how quickly this energy can be stored or released. Very generally, the distinction can be made between faradaic electrochemical processes such as intercalation of ions into the material, which are slow but allow for a high energy density, and non-faradaic physical processes such as double layer formation, which are fast and, therefore, facilitate a high power density. Faradaic processes are intensively studied in the context of battery materials, but origin and contribution of non-faradaic processes, which can be dominant in high surface materials, is still unclear^4^. It was summarized that “for the further advancement of electrical energy storage” systems “the influence of the surface chemistry on the electrochemical properties of materials” needs to be understood^5^. The dynamic interplay between surface and bulk in sense of faradaic and non-faradaic processes or mechanism are therefore of broad interest.

In general the characterization of pristine materials for batteries is of importance to understand their fundamental properties^6,7^. These properties can be tuned by several strategies, like doping with other atoms, by particle size adaption, and by modification of the surface structure. The last two approaches effected different bulk properties for the same bulk material^8^. Therefore the cycling behavior and rate capability in batteries are commonly tested to correlate changes of surface and particle size with changes in the performance^9^.

TiO_2_, for example, is an important, much studied material^10^ that exhibits faradaic and non-faradaic properties, which both depend on particle size and manufacturing process^11,12^. Overall surface modification dependent changes in a bulk material are very difficult to observe. One example of a strong conductivity change in bulk AgI caused by surface modification with Al_2_O_3_ was reported^13^.

Nuclear Magnetic Resonance (NMR) is a low energy spectroscopy method that has extensively been used for the observation of structural features and mobility in battery materials and batteries^14^. Especially longitudinal, or *T*_1_, relaxation time based experiments are capable of characterizing local dynamic processes and are, therefore, valuable methods to elucidate kinetics and equilibria of reaction or transport mechanisms^15^. To extract the maximum amount of information from relaxation experiments, Laplace inversion or inverse Laplace transform (ILT) has been established as a powerful technique^16,17^. More recently, ILT has also been employed for the analysis of *T*_1_ relaxation data of solid battery materials, enabling the assignment of relaxation times to individual contributions and their relative proportions^18^.

In ceramic battery materials it is common to formally charge compensate the insertion or extraction of lithium ions or, more generally, mobile cations with redox processes of immobile transition metals or anions^19,20^. Another possibility to compensate positive charge in solids is presented by mobile, delocalized electrons, which are often referred to as polarons^21^. Polarons are, in a simplified picture, mobile negatively charged quasi-particles with an ionic bond portion^22,23^. The picture of polarons is needed to show that formal negative charge can be distributed over multiple atoms such as titanium and oxygen in a solid like LTO, In such a delocalized form, polarons can significantly contribute to the conductivity of the material. Unpaired electrons or polarons are known to induce faster relaxation on nearby nuclei and it is therefore possible to indirectly detect them via *T*_1_ relaxation measurements^24,25^. Compounds from the binary LiO_2_–TiO_2_ composition line such as, for example, Li_4_Ti_5_O_12_ (LTO) have shown both very good cycling stability and very high rate capability^26,27^. On the other hand, Li ion mobility in stoichiometric LTO appears to be low^28^, yet strongly morphology dependent, with nanostructured LTO showing considerably faster Li ion dynamics^29^. Furthermore, the addition of small amounts of lithium cause the resulting Li_4+X_Ti_5_O_12_ to be a fast conducting material^28,30–32^. Other publications on the diffusion of Li^+^ in rutil^33,34^ and LTO^35^ support the idea of polaron hopping. For LTO, recently developed simulation techniques were able to show that polarons are more stable at the surface than in the bulk^36^. This can also be considered in terms of semiconductor theory and surface potentials^37^.

However, as soon as a material is inserted into an electrochemical cell for testing, a multitude of additional processes occur, which hinders the individual investigation of specific mechanisms. Here, dry LTO is compared to LTO in contact with different electrolytes to analyze potential effects on bulk properties of the solid material. It is investigated whether, in principle, chemical surface reactions are induced, and if these changes affect the Li ion mobility. NMR relaxation is used to identify changes of the dynamics in the material. By employing Laplace inversion of the data, fractional differences in relaxation behavior due to Li mobility in different sample environments can be identified without a priori assumptions regarding the number of individual components. Thereby, it is possible to distinguish between core–shell relaxation behavior if a surface layer changes its properties, and bulk effects if changes on the surface have a long-range impact on the physical properties of the material. The investigations and the resulting changes in the *T*_1_ relaxation times of the solid material lead to the conclusion that there is an equilibrium between the solid material and the electrolyte. The lithium cations from the electrolyte can diffuse into the bulk material, while the anions remain on the surface. This gives the picture of a solid-electrolyte bilayer at the interface. On this basis, we propose a mechanistic relationship between the polaron-induced intercalative pseudocapacitance and the fast-charge capability of LTO.

## Results

**Fig. 1:**
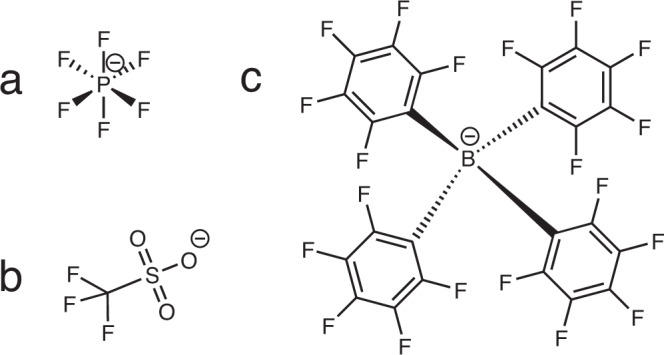
The three anions used in this study. **a** PF_6_^−^, (**b**) Tfl^-^, (**c**) Tpb^−^.

Commercial battery grade nano-LTO powder was brought in contact with different electrolytes (Supplementary Fig. 1) containing ether lithium or sodium as cations and PF_6_^-^, Triflate (Tfl^-^) or Tetrakis-(pentafluorophenyl)-borate (Tpb^−^) as anions (Fig. 1). The longitudinal relaxation time constant *T*_1_ of bulk ^7^Li nuclei was recorded in a temperature range from −10 °C to 50 °C. We inverted the recorded data using ILT without non-negativity constrains^38^. Thereby it is possible to separate relaxation processes in solid lithium conducting materials^18,39,40^.

### Principle of inverse Laplace transformation on the LTO system

**Fig. 2:**
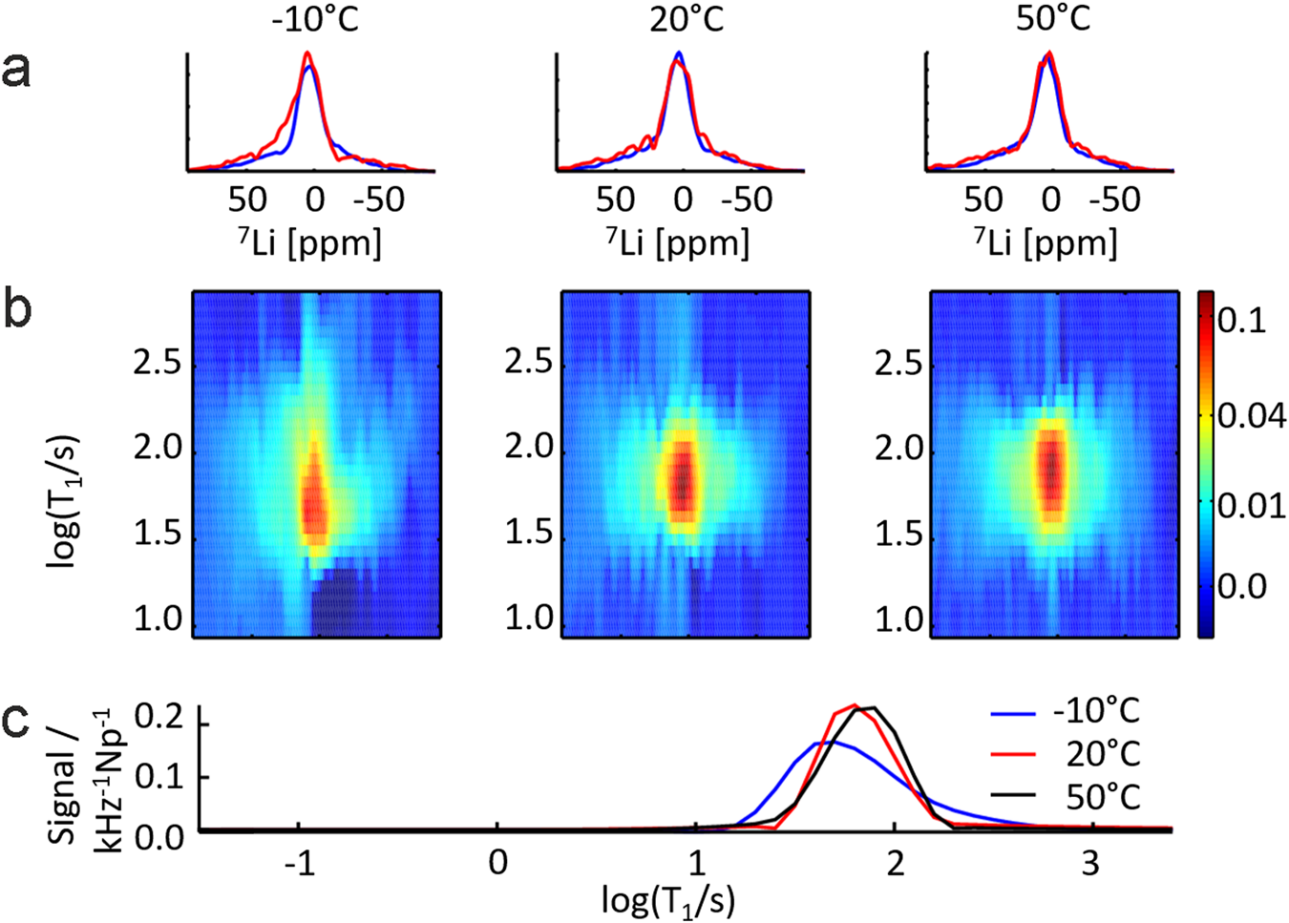
Principle of data analysis demonstrated for static ^7^Li NMR *T*_1_ measurements of nano-LTO powder at −10 °C, 20 °C and 50 °C using inverse Laplace transformation without non-negativity constraint. **a** ^7^Li NMR spectra obtained by integration of the spectrally resolved *T*_1_ distribution between 25 s and 80 s (blue line), and between 100 s and 315 s (red line), scaled by a factor 3. **b** Spectrally resolved distribution of ^7^Li NMR *T*_1_ relaxation time constants. The signal, as indicated by the colorbar, is normalized to the two-dimensional integral of the distribution shown in the panels. The integral is conducted in log space along the *T*_1_ dimension, therefore the unit of the colorbar values is [kHz^-1^Np^-1^], with Np being the unit Neper. **c** *T*_1_ relaxation time distribution of ^7^Li at the maximum of the NMR spectrum.

The procedure is exemplified on pure LTO under Argon atmosphere (Fig. 2). The first row shows static ^7^Li NMR spectra of the sample measured at −10 °C, 20 °C and 50 °C. The blue spectrum is the fast relaxing contribution to the overall spectrum, calculated as the sum of the relaxation distribution between 25 s and 80 s, and the red spectrum is the slowly relaxing contribution to the spectrum between 100 s and 315 s. The second row shows the spectrally resolved *T*_1_ distribution of the three measurements. The third row compares the *T*_1_ distribution of the three temperatures, integrated along the spectral dimension. It is evident that the temperature has a small but observable effect on the maximum and the distribution of the *T*_1_ relaxation. While the exact distribution may vary slightly due to the ill-posedness of the problem, the mean, the median or the width of each mode are quite robust parameters^41^. Hence, the position of the maximum and the mean are used to analyze the effects of the solvents and temperature in the following.

**Fig. 3:**
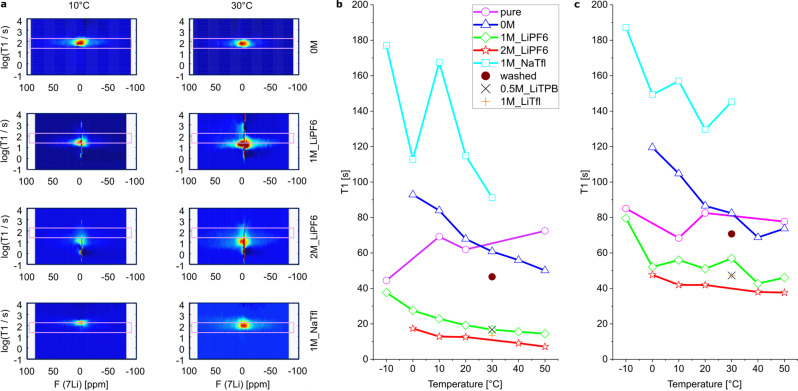
Electrolyte and temperature dependent ^7^Li *T*_1_ relaxation time distributions in LTO. **a** Exemplary *T*_1_ relaxation distribution of ^7^Li in 4 different samples measured at 10 °C and 30 °C (**b**) Position of maxima and (**c**) mean distribution of *T*_1_ relaxation of ^7^Li in all 8 LTO samples with changing electrolyte. No electrolyte (pink circle, pure), organic solvent without salt (blue triangle, 0 M), 1 M LiPF_6_ electrolyte (green diamonds, 1M_LiPF6), 2 M LiPF_6_ electrolyte (red stars, 2M_LiPF6), 1 M NaSO_3_CF_3_ (cyan squares, NaTfl), 1 M sample washed and measured with organic solvent without salt (brown dot, washed), 0.5 M LiC_24_BF_20_ (black X, 0.5M_LiTPB), 1 M LiSO_3_CF (orange cross, 1M_LiTfl).

We prepared a series of 8 different samples (Fig. 3). The results of the ILT for four samples measured at 10 °C and 30 °C is given exemplary in Fig. 3a. A pink box is drawn as guide for the eye. It marks the upper and lower *T*_1_ relaxation values for the LTO in EC/DMC at 30 °C as reference (Fig. 3a, first row). The use of 1 M LiPF_6_ (Fig. 3a, second row) and 2 M LiPF_6_ (Fig. 3a, third row) result in a reduction of the bulk relaxation. The use of 1 M NaTfl (Fig. 3a, last row) shows the opposite effect. It is noteworthy that the lithium containing electrolytes cause an additional liquid-state ^7^Li NMR signal with a relaxation time of about 2 s. It originates from solvated Li^+^ in the electrolyte and can easily be separated in the relaxation time dimension. However, subtraction of this feature by using the pure electrolyte is not straightforward since weak features, most probably caused by Li ion exchange between electrolyte and LTO, lead to spectrally narrow signal components at relaxation times of Li ions in the solid. This is expected if Li ions exchange between the inversion pulse and the detection pulse, hence relax during part of the recovery delay in one environment and are detected in the other.

We extract from these results in the aforementioned way the maxima distribution (Fig. 3b) and the mean distribution (Fig. 3c) of the *T*_1_ relaxation of each sample at a given temperature.

### Results of inverse Laplace transformation on the LTO system

Pure LTO under Argon atmosphere (Fig. 3b, c, pink circles) shows at -10 °C the lowest value in the *T*_1_ maxima distribution at 44.5 s and a highest value of 72.4 s at 50 °C. It is the only sample, where the maximum of relaxation time rises with increasing temperature. The Mean distribution stays quite constant between 70 s and 85 s for the same sample (Fig. 3b, c, pink circles). Such a convergence of the maximum and mean indicates that the relaxation mode becomes more symmetric at increased temperatures, which can also be observed in Fig. 2c.

The contact with an organic solvent consisting of dimethylcarbonate and ethylcarbonate (1:1 by volume), henceforth called electrolyte, without salt (0 M, blue triangles, Fig. 3b, c) resulted at 20 °C in similar relaxation behavior as the dry material. It shows a change in the slope of the temperature dependence of the maximum distribution and the mean distribution. For both distributions of the 0 M sample an increase in temperature results in a decrease in relaxation time (blue line, Fig. 3). Yet the effect is weak and will not be further discussed.

We used a 1 M LiPF_6_ (1M_LiPF6, green diamonds, Fig. 3b, c) solution with the same organic solvent as next eluent. It resulted in a significant reduction of the *T*_1_ relaxation time of the bulk ^7^Li signal for all temperatures. The maximum of the *T*_1_ relaxation time distribution at 30 °C drops from 60.8 s in 0 M to 16.7 s in 1M_LiPF6 sample, while the mean value drops from 82.4 s to 56.9 s. This is a general trend for all temperatures. The effect increases when going to a 2 M LiPF_6_ solution (2 M, red stars, Fig. 3). The relaxation time value at 30 °C is 10 s for the maximum and 40 s for the mean value.

Such a strong relaxation effect, which affects the whole *T*_1_ distribution of ^7^Li in LTO, is only plausible if Li in the LTO bulk is affected by a changing mobility or an altered strength of interactions responsible for relaxation, such as additional paramagnetic centers in the bulk. Additional mobile electrons in the bulk material could explain it. But their origin needs to be further addressed. It was shown that the small amount of additional lithium (Li^+^ + e^−^) in Li_4.1_Ti_5_O_12_ caused a tremendous reduction in *T*_1_ relaxation time of ^7^Li and with this an increased mobility of lithium ions, due to more mobile electrons in the bulk material^28^.

To check whether the PF_6_^−^ anion has specific properties that cause this effect, we compared the results with LiTfl and LiTpb, which are two chemically very different electrolyte salts with non-coordinating anions (Fig. 1). All three anions are very weakly coordinating and have only the fluorine as element in common. Still the effect on the ^7^Li relaxation time was reproducible and seems therefore to be independent of the used anion (Fig. 3b, c black X and orange cross).

To further assure this effect is not induced by an irreversible surface modification e.g. due to the introduction of fluorine, we decantively washed the 1 M sample of LiPF_6_ four times with the salt-free organic solvent. The resulting sample (washed, brown dot, Fig. 3) had strongly elongated relaxation time, close to the 0 M sample. It shows the effect is at least to a major part reversible and, therefore, excludes irreversible surface modifications on the LTO nano particles to be responsible^42^. Any additional mobile negative charges must therefore be from LTO itself.

To investigate the role of the lithium cation we measured a 1 M solution of sodium triflate (NaTfl, bright blue line, Fig. 3) between −10 °C and 30 °C. Since sodium cannot intercalate into LTO. The mean of the relaxation time distribution increased at least by 20 s, which again can only be explained if relaxation of Li ions in the bulk of LTO is affected. The maxima of the relaxation show a larger deviation around the trend line, which is caused by the logarithmic sampling of the relaxation times during measurement. Nevertheless, one can adjust the maximum bulk relaxation time *T*_1_ in those LTO nanoparticles within one order of magnitude using lithium or sodium containing electrolytes.

In the electrolyte free lithium cations could be observed, which are probably caused by a partial exchange of Li with Na in LTO. Such an exchange could lead to a stronger shifting of polarons to the LTO surface. Since Na is restricted to the LTO surface only, initially bulk polarons, which enable the mobility of lithium cations in the bulk, now lead to a reduced influence on the bulk relaxation time *T*_1_. With lithium containing electrolytes lithium ions from the solution diffuse into the bulk material, acquiring polarons from the surface.

## Discussion

**Fig. 4:**
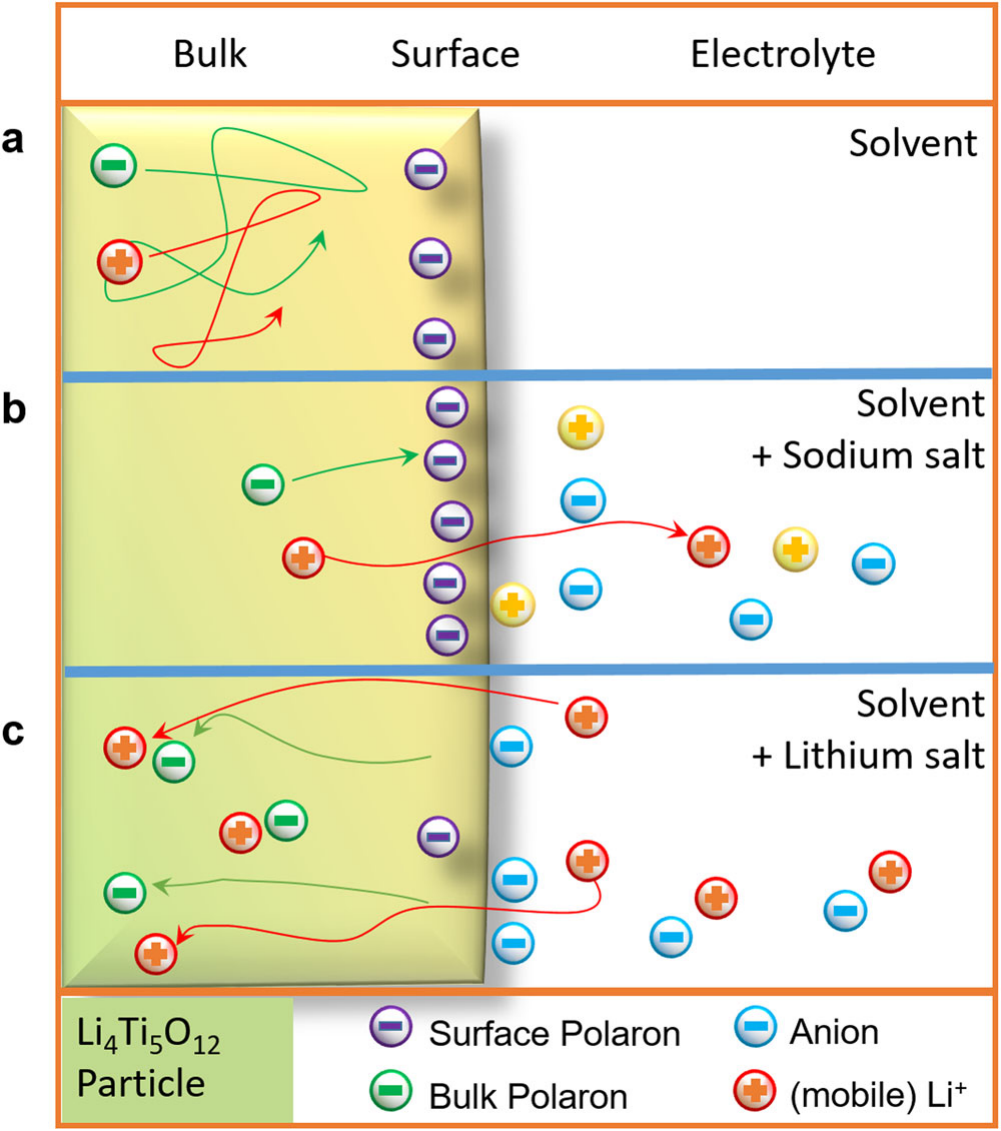
Equilibria between electrolyte, surface and bulk of LTO. The electrolyte DMC/EC has minor effect on the surface-bulk equilibrium of polarons (**a**). Sodium cations interact with the surface and shift polarons to it (**b**). Lithium cations from the electrolyte can diffuse with the surface polarons into the bulk: Anions are forced to interact with the surface (**c**).

From these results we can conclude on the equilibria in the “three phase system” of LTO bulk, LTO surface and electrolyte (Fig. 4). There are three cases to be summarized. First, an organic solvent has only a marginal effect on the equilibrium between surface and bulk. It leaves the equilibrium from that point of view untouched (Fig. 4b). Second, a sodium containing electrolyte causes a shift of the mobile polarons from the bulk to the surface, as mobile lithium ions can exchange with sodium ions from the electrolyte. As the mobility of lithium in LTO is correlated with a mobile polaron, this negative charge is now pinned to sodium on the surface. (Fig. 4a). Finally a lithium containing electrolyte will cause a shift of polaron concentration from the surface into the bulk (Fig. 4c). Excess lithium cations from the solvent can diffuse into the bulk and drag the polarons due to charge neutrality in bulk with them. The Anions should only have a minor influence on this polaron concentration shift. They are very weakly coordinating, and in case of Tpb^-^ the negative charge is sterically very well shielded.

This picture of the equilibria is supported by additional experiments. ^19^F spectra and *T*_1_-relaxation data of LTO with low concentrations of LiTFSI and NaTFSI in DMC were recorded and are shown in supplementary information (Supplementary Note 1). They show selective anion-surface interaction in case of the Lithium salt, but no interaction in case of the sodium salt (Supplementary Note 2, 3; Supplementary Fig. 2–8). This means lithium diffuses from the electrolyte into the bulk and the anions are forced to interact with the surface for charge compensation, while the anions of the sodium electrolyte do not feel this force. We estimate the amount of adsorbed anions from a LiTFSI solution on LTO to be about one anion per 550 molecular formulae of Li_4_Ti_5_O_12_ (Supplementary Note 4). Furthermore an Arrhenius plot of the *T*_1_ data (Supplementary Note 5; Supplementary Fig. 9) shows a decreasing of the slope when going from 0 M to 1 M to 2 M. This can qualitatively be interpreted as a change from a ^7^Li NMR relaxation mechanism that is dominated by nuclear dipole–dipole interactions towards relaxation caused by paramagnetic species in the vicinity of the nuclear spins. It was shown the *T*_1_ relaxation in Li_4+X_Ti_5_O_12_ (X = 0) changes from a nuclear dipol-dipol relaxation to a paramagnetic relaxation in Li_4+X_Ti_5_O_12_ (X = 0.1)^28^. The Lithium stoichiometries in the bulk of M1 and M2 lie between those points (0 > X > 0.1) and therefore show the transition from dipol-dipol to paramagnetic dominated relaxation. This fortifies additional negative charges cause the observed relaxation changes and this supports again the picture of surface polaron mobilization. Additionally electrochemical impedance spectra of dry LTO powder and dry LTO powder with adsorbed LiTFSI are given (Supplementary Note 6; Supplementary Fig. 10).

**Fig. 5:**
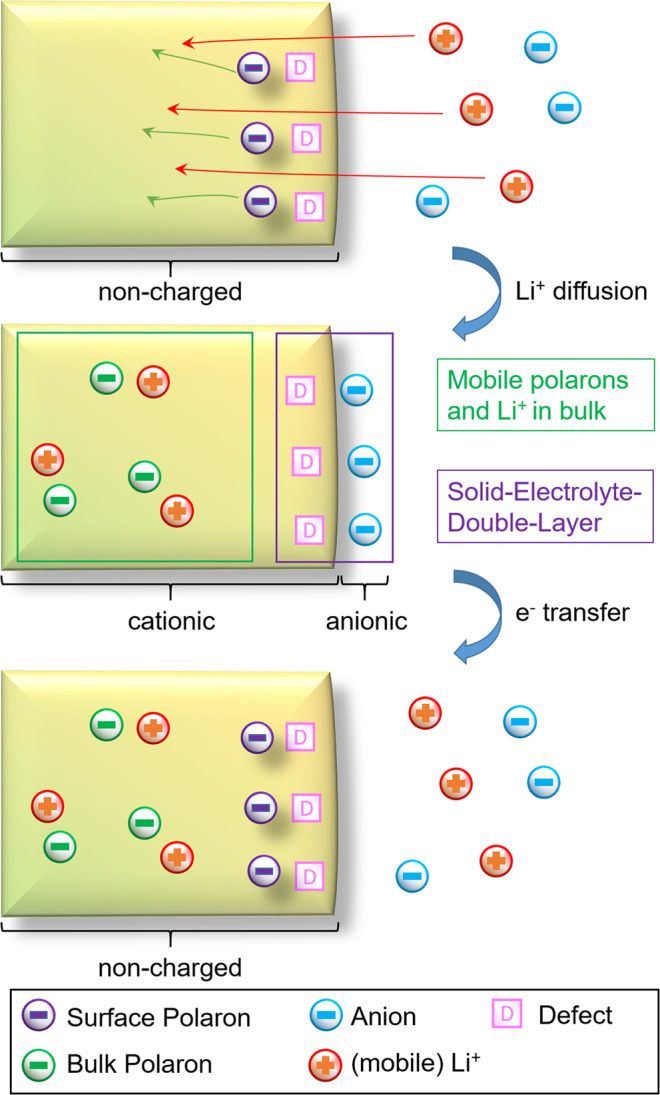
Surface defects with polarons enable lithium cations to diffuse into the bulk Li_4_Ti_5_O_12_. It results a Solid-Electrolyte-Double-Layer to compensate the now positive charged particle due to the additional lithium cations in the bulk. A subsequent electron transfer to the particle restores a neutral Li_4+x_Ti_5_O_12_ particle. X being the equivalent of intercalated lithium atoms.

Following up the point of charge neutrality, which is very important for a complete picture of the equilibrium between solid and electrolyte. The overall LTO nano-particles are not charged when in contact with the pure organic solvent. The lithium containing electrolyte causes additional positive charge carriers into the material by inserting lithium cations. Their positive charges are compensated in the bulk by the mobile polarons. The introduced positive charge are compensated by the anions, which form a layer close to the defects or vacancies on the LTO surface, leading to a solid electrolyte double layer (Fig. 5). This process could be described as intercalative adsorption process, where the lithium ions intercalate into the material and the respective anions adsorb onto the surface. This fits to the observation of adsorbed LiPF_6_ salts on soaked LTO electrodes^42^ and could contribute to the discussion about SEI formation on LTO^43–48^.

Furthermore, we can conclude on a polaron based charge storage mechanism in LTO materials. It shows the surface with its defects and polarons does have a great influence on the electrochemical properties of the material. It can help to explain the observed high charging rates of very high surface LTO materials, with different capacitance contributions. In such systems it is possible that lithium diffusion into the material has happened before any electrons are transferred to the LTO. Then lithium diffusion into the material is not rate determining anymore, but the electron transfer is the rate determining step during the charging process. So when an electron is transferred to LTO, it compensates only a charge from the anion attached to the surface, making it a very fast process, like in a capacitor, enabling C/N > 300^29,49^. When on the other side the lithium diffusion process is limiting, due to a lower surface area and fewer polarons, the charging process is less effected by the “preequilibrium”, resulting in a more faradaic storage mechanism.

## Conclusion

It was observed that LTO changes its bulk properties by contact with electrolyte in the form of different ^7^Li NMR *T*_1_ relaxation times. This effect is caused on the one hand by the polarons of surface or near-surface defects and on the other hand controlled by the cations of the electrolyte.

On the chemical level, an equilibrium between the liquid and solid phases can be concluded. Lithium ions from the electrolyte can diffuse together with the polarons from the surface into the bulk material. Thus - with respect to a charging process - the ion transport and the electron transport in LTO initially take place separately.

Consequently, the here presented work demonstrates the significant effect of surface defects and the associated polarons, on the properties of LTO as lithium storage materials. Hence, we believe the control of surface defects and polarons is an important tool to tune materials like LTO for battery or supercapacitor application. We propose that other materials of systems such as sodium and potassium ion batteries may be optimized for fast charging as well, if the materials show an intercalative adsorption of the respective electrolytes^50,51^. Furthermore it will be interesting to investigate LTO within the emerging topic of dual-ion batteries^52^. Such high voltage cells may be able to tap the full potential of LTO in fast charging applications^53^.

## Methods

All chemicals were purchased from Merck and stored under Argon atmosphere in a glovebox. Electrolytes and organic solvents were used without further purification. LTO nanopowder (<200 nm) and lithium salts were stored for at least one week in a glovebox before use to remove potential water impurities.

### Sample preparation

For each experiment a Young-type NMR tube 150 mg of LTO was filled and 1 mL of electrolyte was added. The electrolyte consists of EC/DMC (1:1 weight ratio) mixture, having a 0 M, 1 M or 2 M concentration of LiPF_6_, LiTfl, LiTpb or NaTfl. The tube was shaken until a homogeneous slurry was obtained. Measurements were performed after 24 h of settlement. A picture of a typical sample is in the supplementary information (S1).

### NMR measurement

Static NMR experiments were performed on a Bruker Avance III HD 600 wide-bore spectrometer equipped with a gradient probe (Bruker PA BBO 600W2/S4 BB-H&F-D-05 DIFF). Saturation recovery experiments were used to determine ^7^Li *T*_1_ relaxation time. The number of accumulations was 8, using pi/2 pulses of 15 µs at 50 W. A series of up to 38 delays were used from 0.00001 s to 1500 s determine the buildup of the ^7^Li signal.

### Inverse Laplace transformation

Relaxation data was inverted using uniform penalty regularization^16^, which is a Tikhonov regularization algorithm in generalized form, in combination with a zero-crossing penalty to prevent sign changes in the calculated distribution that are not supported by the data^38^. Analyses were done with GNU Octave (version 4.0.3) using a home-written ILT function. For inversion, the 2D relaxation vs. spectral data was used. Thereby, only the relaxation dimension was inverted, but both dimensions were regularized. The resulting sensitivity gain from regularization along the non-inverted spectral dimension facilitated an improved resolution in the inverted relaxation dimension^40^. The regularization parameters have been chosen as detailed in^54^. The *T*_1_ distribution was calculated at logarithmically spaced time constant values between 10^–5^ s and 10^8^ s, with 10 points per decade. The upper limit was chosen to fit the bias, which occurs in saturation recovery experiments, into the distribution rather than as a single value with nominally infinite time constant. It was found empirically that this procedure produces more robust results in the case of multidimensional data sets.

## Supplementary information

supporting information

## Data Availability

The data that support the findings of this study are enclosed in the supplementary material or are available from the corresponding author upon reasonable request.
