# Supplementary material for: Electrolyte contact changes nano-Li4Ti5O12 bulk properties via surface polarons
Source: Commun Chem. 2023 Jun 7;6:113. doi: 10.1038/s42004-023-00913-6 (PMC10247714; doi:10.1038/s42004-023-00913-6)
Supplement: Supplementary file 1 — supporting information [file 42004_2023_913_MOESM1_ESM.pdf]

*Supporting Information*

# Electrolyte contact changes nano-$Li_4Ti_5O_{12}$ bulk properties via surface polarons

P. Philipp M. Schleker*, Cristina Grosu, Marc Paulus, Peter Jakes, Robert Schlögl, Rüdiger-A. Eichel, Christoph Scheurer, Josef Granwehr

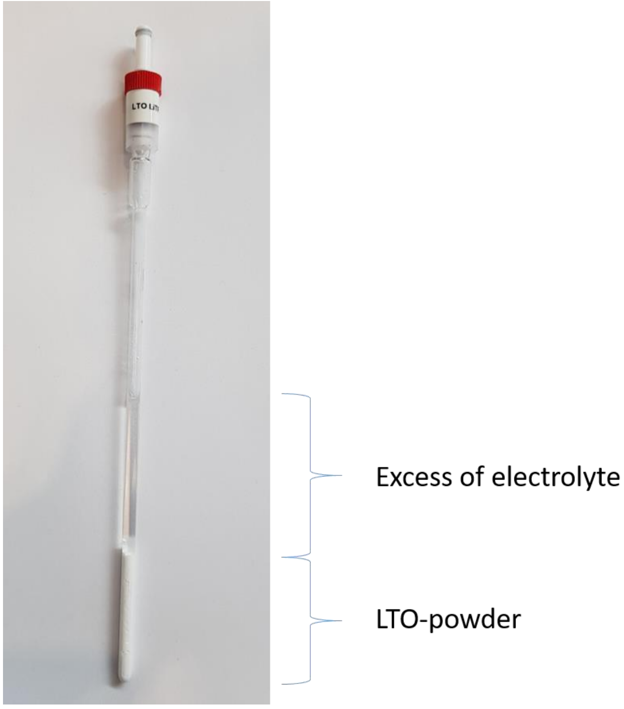


**Supplementary Figure 1**: Image of a typical sample with settled LTO powder and excess of electrolyte in an airtight Young type NMR tube.

## Supplementary Note 1: Anion–surface interaction

The solid–electrolyte equilibrium between LTO and lithium bis(trifluoromethanesulfonyl)imide (LiTFSI) and sodium bis(trifluoromethanesulfonyl)imide (NaTFSI) was investigated. 1.5mL of stock solution containing either the sodium salt [0.69mg; 2µmol] or the lithium salt [1.15mg; 4µmol] in dimethyl carbonate (DMC) were added to 100mg of LTO. After shaking and settling for 24h hours, $^{19}$F NMR spectra were acquired.

## Supplementary Note 2: Sodium bis(trifluoromethanesulfonyl)imide (NaTFSI)

In case of the sodium salt, a small shift of 0.09ppm (49Hz) is observed when comparing the stock solution with the LTO–stock solution mixture (Supplementary Fig. 2). The $T_1$ relaxation time constant shows only a minor difference between both samples, suggesting a surface interaction independent mobility. Even a further measurement of the frozen sample at –10°C did not reveal an additional peak for a surface adsorbed species (Supplementary Fig. 3). Further analysis of the $^{19}$F $T_1$ relaxation data by Inverse Laplace Transformation supports the result of hardly adsorbed species by a main single relaxation time (Supplementary Figs. 4, 5).

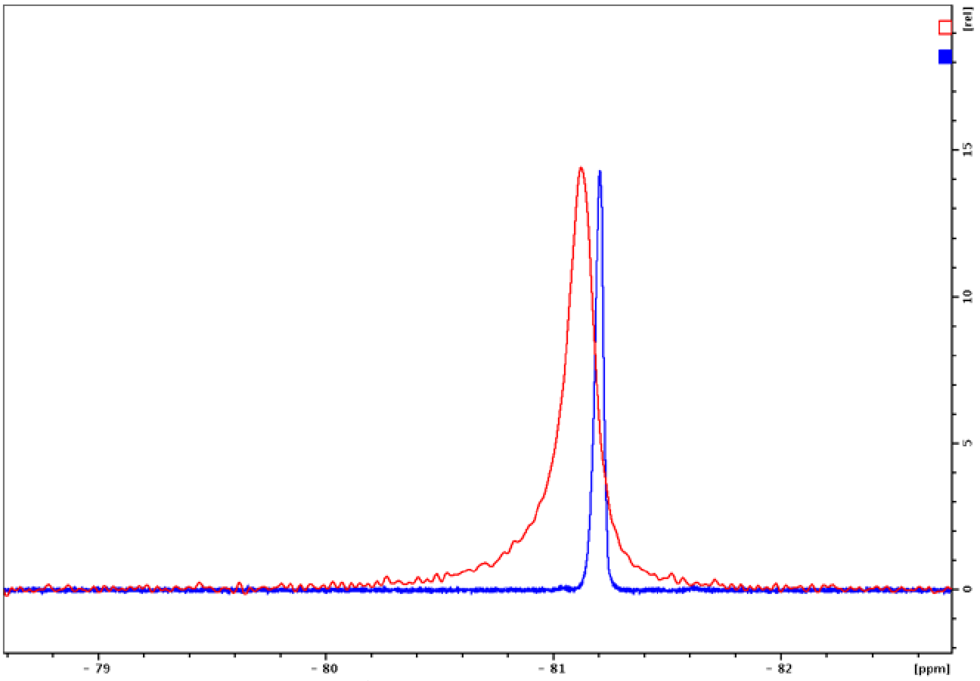


**Supplementary Figure 2**: $^{19}$F NMR spectrum of NaTFSI in DMC (stock solution) measured at 12°C (blue), and LTO plus 2µmol NaTFSI in 1.5mL DMC (red).

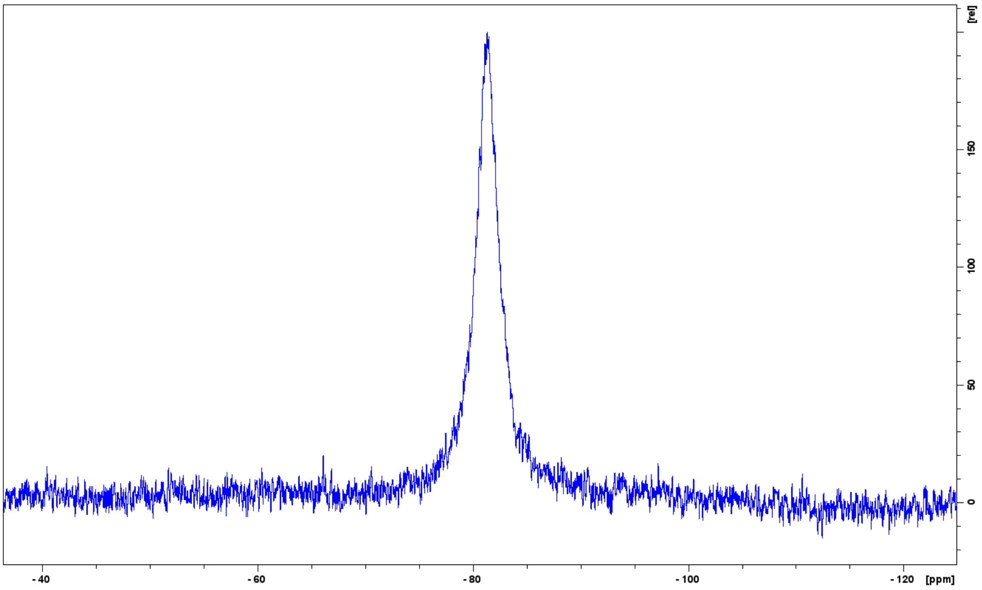


**Supplementary Figure 3**: $^{19}F$ solid echo NMR spectrum of the frozen solution. LTO plus 2µmol NaTFSI in DMC measured at –10°C.

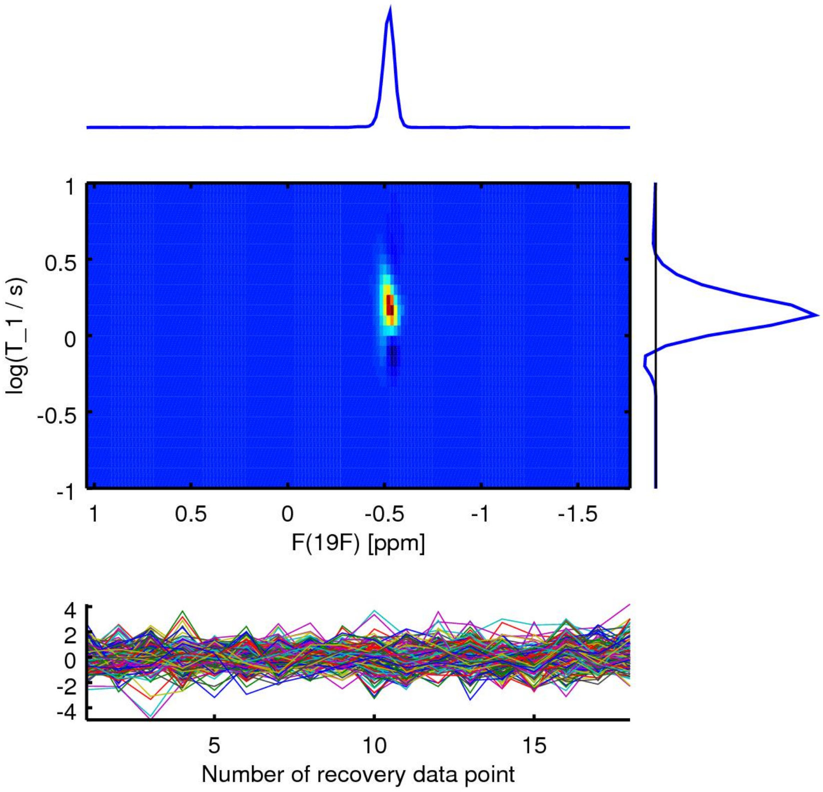


**Supplementary Figure 4**: (top) Inverse Laplace Transformation of $^{19}F$ $T_1$ relaxation data of NaTFSI in DMC (stock solution) *vs.* $^{19}F$ NMR frequency, measured at 12°C. (bottom) Residuals of fit, normalized to root mean square of thermal electronic noise.

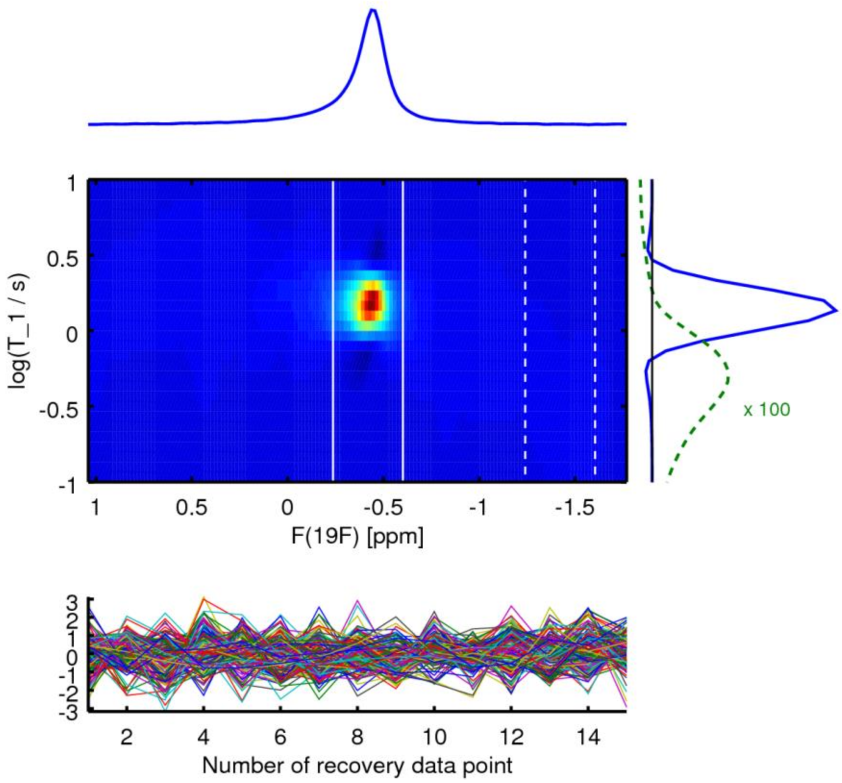


**Supplementary Figure 5**: (top) Inverse Laplace Transformation of $^{19}$F $T_1$ relaxation data of LTO plus 2µmol NaTFSI in DMC *vs.* $^{19}$F NMR frequency, measured at 12°C. (bottom) Residuals of fit, normalized to root mean square of thermal electronic noise.

**Supplementary Note 3: Lithium bis(trifluoromethanesulfonyl)imide (LiTFSI):**

In case of the lithium salt, a $^{19}F$ NMR shift of 0.18ppm (103Hz) is observed and additionally a second, broader component with a shift of 0.66ppm (374Hz) is clearly visible (Supplementary Fig. 6). These signals are temperature dependent. The signals start to merge with increasing temperature, proving exchange between two reservoirs – surface-interacting anions and anions of the inter-LTO-particle solution. The broader component has a shorter $T_1$ value, suggesting a stronger interaction with the surface, which supports the charge compensation on the solid (Supplementary Fig. 8)
This proves the picture of a double layer that is formed when using lithium salts, and not for sodium salts. This is a further demonstration of the selective intercalation of lithium cations into the material.

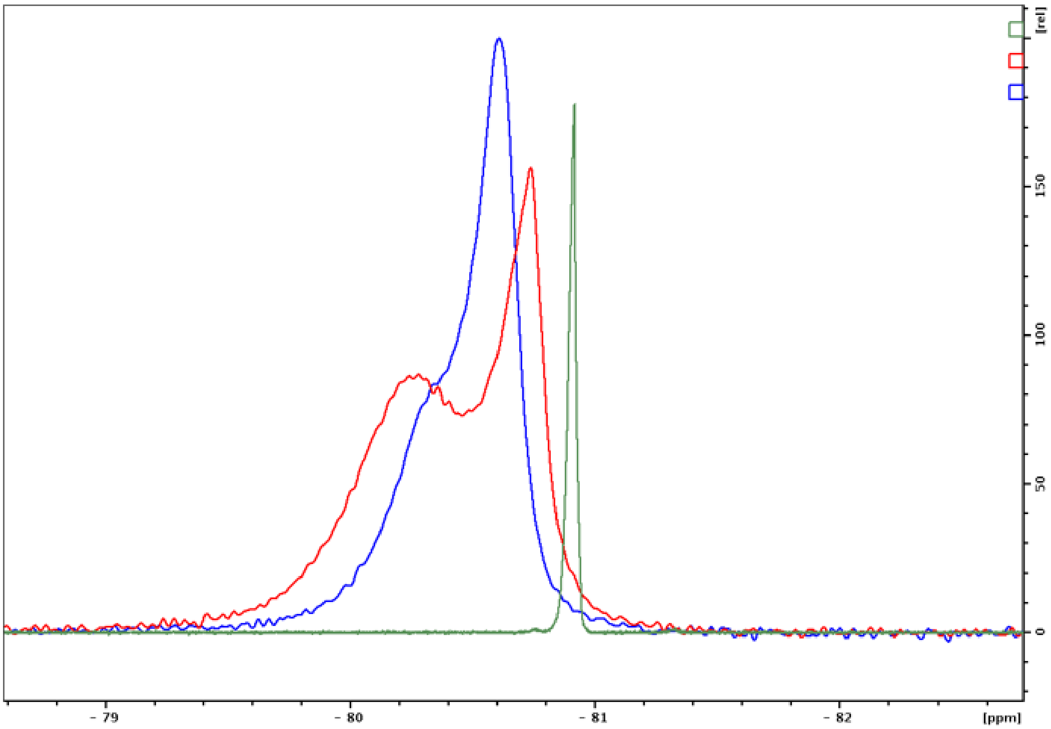


**Supplementary Figure 6**: $^{19}F$ NMR spectra. (green) LiTFSI in DMC (stock solution) measured at 12°C. (blue) LTO plus 4µmol LiTFSI in 1.5mL DMC measured at 22°C. (red) LTO plus 4µmol LiTFSI in 1.5mL DMC measured at 12°C.

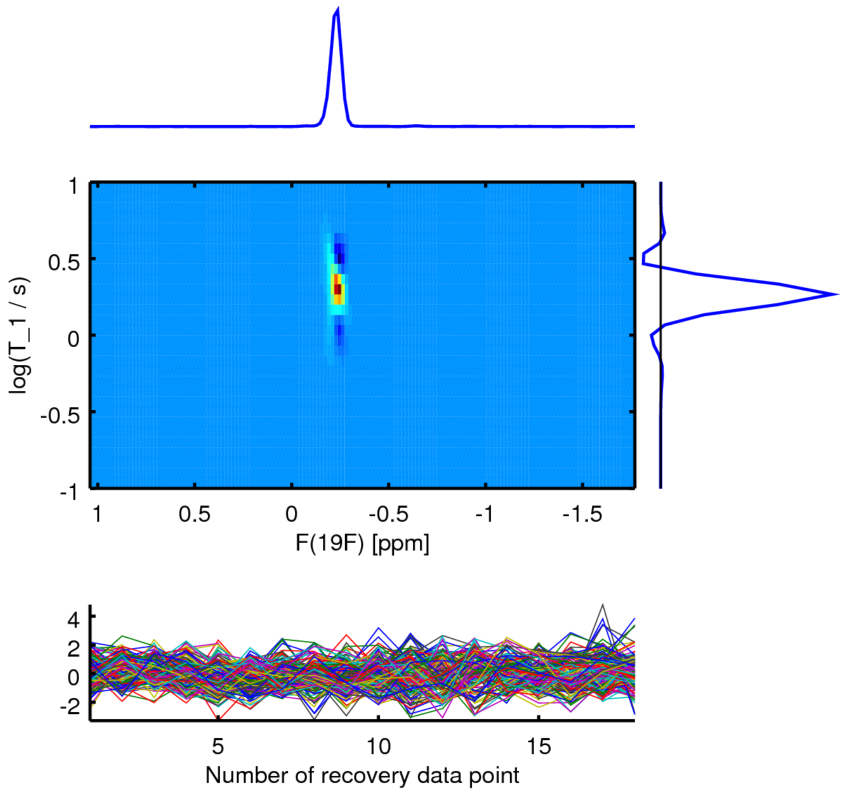


**Supplementary Figure 7**: (top) Inverse Laplace Transformation of $^{19}F$ $T_1$ relaxation data of LiTFSI in DMC (stock solution) *vs.* $^{19}F$ NMR frequency, measured at 12°C. (bottom) Residuals of fit, normalized to root mean square of thermal electronic noise.

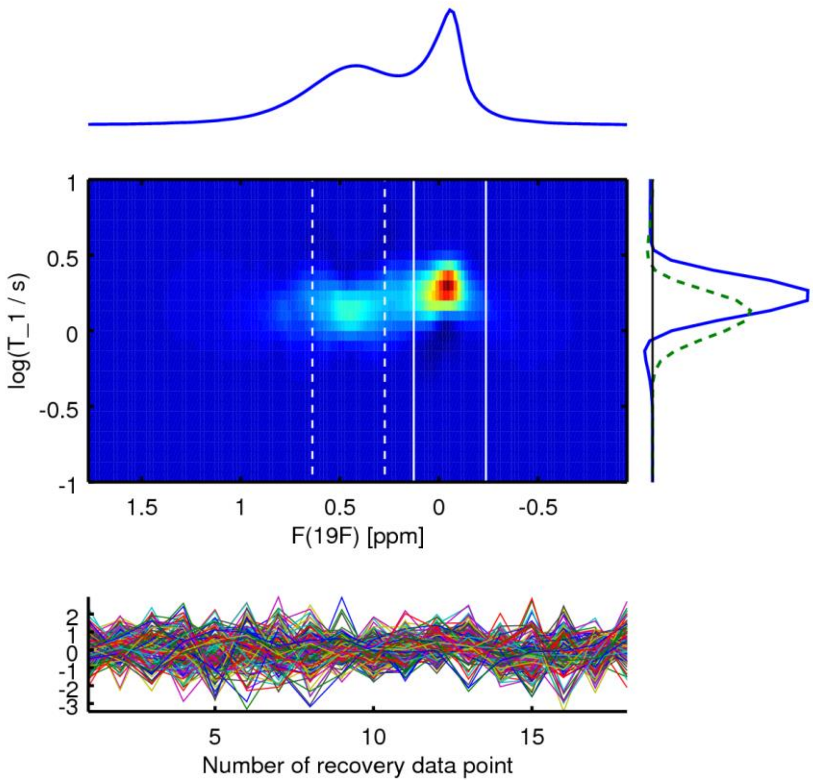


**Supplementary Figure 8**: (top) Inverse Laplace Transformation of $^{19}F$ $T_1$ relaxation data of LTO plus 4µmol LiTFSI in DMC *vs.* $^{19}F$ NMR frequency, measured at 12°C. (bottom) Residuals of fit, normalized to root mean square of thermal electronic noise.

**Supplementary Note 4**: **Estimation of polaron concentration**

The concentration of polarons was estimated by quantitative NMR of the adsorbed anions.

The amount (*n*) of 4µmol LiTFSI was dissolved in 1.5mL DMC (Volume A) and was filled in a Young type NMR tube. This volume corresponds to a height ($H_A$) of 10.4cm in the Young type NMR tube. A quantitative $^{19}$F NMR spectrum was recorded.
100mg LTO was added, shaken and waited until the powder was settled (24h). The volume increased to a height of 10.6cm in the NMR tube. The settled LTO (Volume B) had a height ($H_B$) of 3.9cm in the tube and the supernatant solvent (Volume C) had a height ($H_C$) of 6.7cm. The integrals $I_A$, $I_B$, $I_C$ of the quantitative $^{19}$F NMR spectra of volumens A, B, and C were determined, with $I_A$, $I_B$, $I_C$ proportional to the concentration of fluorine nuclei in the detection volume. Volumes B and C could be distinguished by shifting the position of the NMR tube such that the volume of interest resided within the coil used for $^{19}$F NMR detection. The coil had a hight on the order of 1 cm, allowing for a differentiation of the two volumes.

The amount $\gamma_X$ of anions (in an artificial unit of cm) in a volume X can be correlated to the signal intensity $I_X$ and height $H_x$ of that volume in the NMR tube,

$$\gamma_X = I_X H_X$$

The amount of anions in A should be the same as in B+C combined, since no additional fluorine was provided when LTO was added. We expect

$$I_A H_A = I_B H_B + I_C H_C$$

The concentration of anions in solution increased in B and decreased in C in comparison to A. With the measured values for the integrals ($I_A$ = 81.4, $I_B$ = 103, $I_C$ = 70.5, in relative arbitrary units), both sides should give the same value:

$\gamma_A$ = 81.4 x 10.4 cm ≈ 847 cm
$\gamma_B$ + $\gamma_C$ = 103 x 3.9 cm + 70.5 x 6.7 cm ≈ 874 cm

The discrepancy is
(874 cm/ 847 cm)–1 = 0.032, or 3.2%

Such an error is within the range expected with a measurement error of ±0.1 mm for $H_X$, hence the measurements of the relative amounts are sufficiently quantitative to draw conclusions on the amount of additional anions ($\delta$) in volume B, which corresponds to the amount of anions adsorbed by LTO.
$\delta$ = ($I_B$–$I_A$)*3.9 cm = (103 – 81.4)*3.9 cm = 83.4 cm

By this the total amount of anions ($n_{adsorbed}$) adsorbed by LTO can be calculated as
$n_{adsorbed}$ = $n$ $\delta$ / $\gamma_A$ = 4 µmol * 83.4 cm / 847 cm = 0.39 µmol

Assuming the adsorption is mainly driven by the intercalative adsorption process, each anion corresponds to a polaron in LTO. With this we can estimate to have about 0.39 µmol polarons in 100mg, or 217 µmol, of $Li_4Ti_5O_{12}$ nano powder, corresponding to 1 polaron every 550 molecular formulae of $Li_4Ti_5O_{12}$.

## Supplementary Note 5: Arrhenius plot of relaxation data

Arrhenius plot representation of the $^7$Li $T_1$ relaxation data (Figure S9). Although the temperature range for the measurements of the two-phase system was limited, some important conclusions can be drawn. The $T_1$ measurements of a partially lithiated LTO material (such as $Li_{4.1}Ti_5O_{12}$) can be interpreted in terms of the Curie–Weiss relaxation as paramagnetically induced relaxation caused by mobile polarons in the bulk material. In case of an unlithiated sample, polarons are barely present in the bulk, and dipol–dipol or quadrupolar coupling induced relaxation are expected to be the main relaxation pathways. The decreasing slope from 0M to 1M to 2M is, therefore, best interpreted as transition from a dipol–dipol/quadrupolar relaxation dominated process to a process with paramagnetic as well as dipol–dipol/quadrupolar contributions. A credible calculation of an activation barrier is therefore not possible, but the qualitative statement that additional negative charges in the bulk cause the observed changes in relaxation rate is supported.

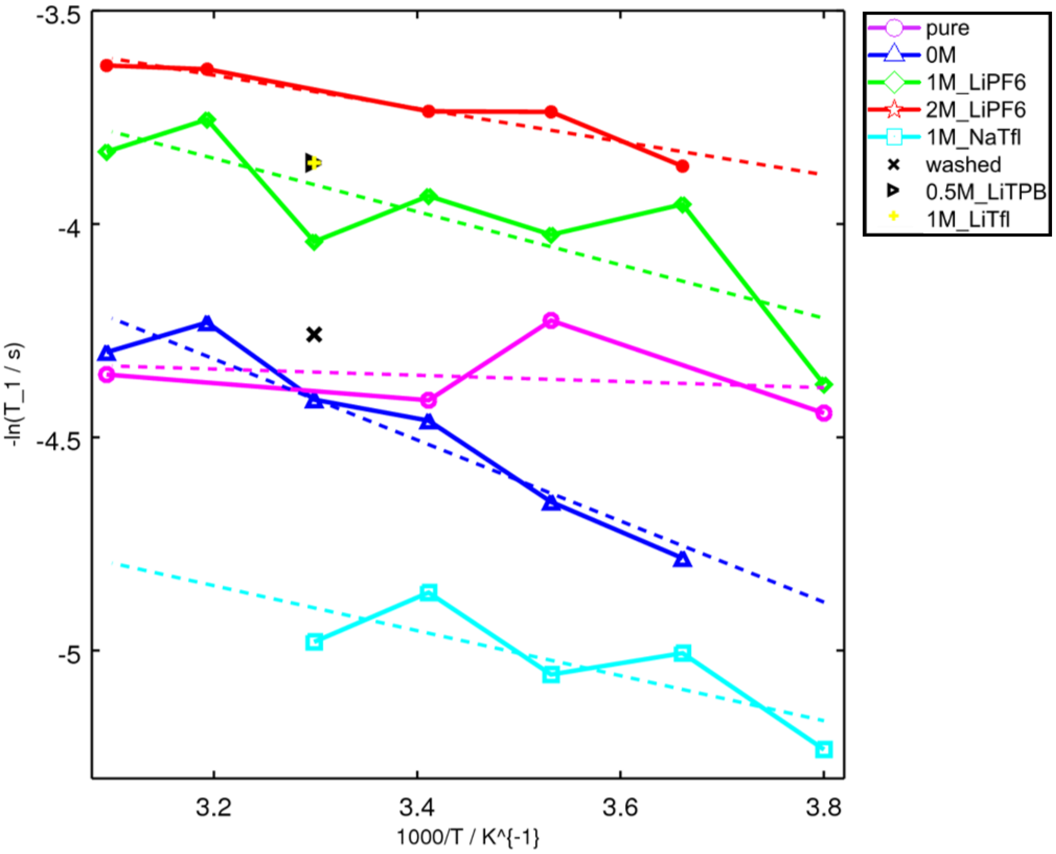


**Supplementary Figure 9**: Arrhenius plot representation of the $^7$Li $T_1$ relaxation data (mean of distribution). No electrolyte (pink circle, pure), organic solvent without salt (blue triangle, 0M), 1 M $LiPF_6$ electrolyte (green diamonds, 1M_LiPF6), 2 M $LiPF_6$ electrolyte (red stars, 2M_LiPF6), 1 M $NaSO_3CF_3$ (cyan squares, NaTfl), 1M sample washed and measured with organic solvent without salt (black X, washed), 0.5 M $LiC_{24}BF_{20}$ (black triangle, 0.5M_LiTPB), 1 M $LiSO_3CF$ (yellow cross, 1M_LiTfl).

## Supplementary Note 6: Electrochemical impedance spectra

1.5mL of stock solution containing lithium bis(trifluoromethanesulfonyl)imide (LiTFSI) [1.15mg; 4µmol] in dimethyl carbonate (DMC) were added to 100mg of LTO. The sample was dried under vacuum to receive LTO with potentially adsorbed LiTFSI (called LTO+Li(TFSI)).
A sample of 10mg pure and dry LTO powder (called LTO_pure) is compared to a sample of LTO+Li(TFSI). Electrochemical impedance spectra (EIS) were performed in a Swagelok cell between two steel stamps on the potentiostat VSP-300 from Bio-Logic. A frequency range from 1MHz to 0.5Hz at a temperature of 22°C was used.
The EIS measurement shows a tendency of faster components in the case of LTO+Li(TFSI). This is part of ongoing work.

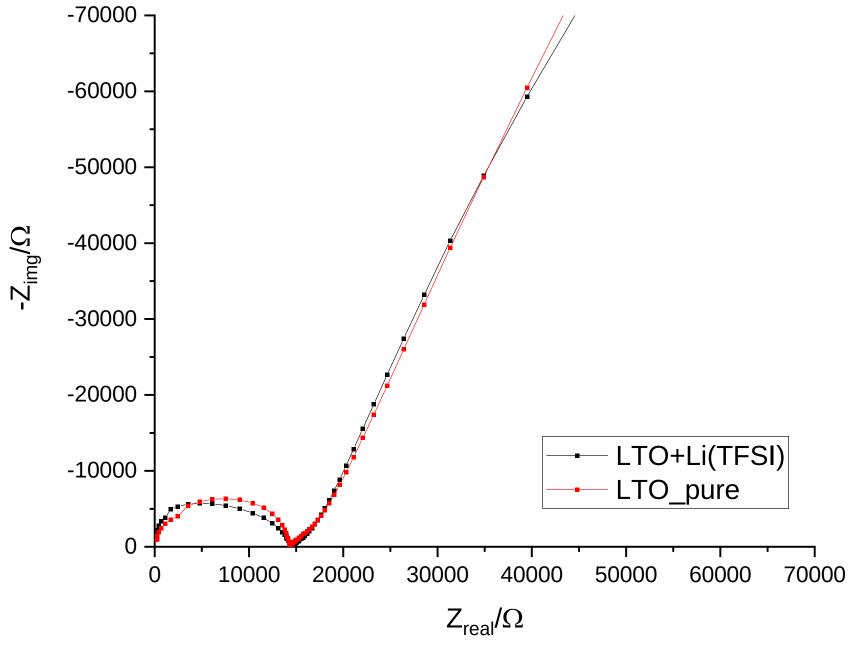


**Supplementary Figure 10**: Electrochemical impedance spectra of dry LTO powder (red) and dry LTO powder with adsorbed LiTFSI (black).
